# Synthesis, structure, and fluorescence properties of a calcium-based metal–organic framework†

**DOI:** 10.1039/c8ra06043f

**Published:** 2018-09-10

**Authors:** Daichi Kojima, Tomoe Sanada, Noriyuki Wada, Kazuo Kojima

**Affiliations:** Department of Applied Chemistry, College of Life Sciences, Ritsumeikan University 1-1-1 Noji-Higashi Kusatsu-City Shiga 525-8577 Japan kojimaka@sk.ritsumei.ac.jp; Department of Materials Science and Engineering, National Institute of Technology, Suzuka College Shiroko-Cho Suzuka-City Mie 510-0294 Japan

## Abstract

The solvothermal reaction of a mixture of calcium acetylacetonate and 1,4-naphthalenedicarboxylic acid (H_2_NDC) in a solution containing ethanol and distilled water gave rise to a metal–organic framework (MOF), {(H_3_O^+^)_2_[Ca(NDC)(C_2_H_5_O)(OH)]}_4_·1.1H_2_O. This MOF possesses a new structure composed of calcium clusters and H_2_NDC linker anions and shows a unique fluorescence property; it exhibits a fluorescence peak at 395 nm (*λ*_ex_ = 350 nm) at room temperature, which is blue-shifted compared with that exhibited by the free H_2_NDC ligand. One of the possible mechanisms for this fluorescence is likely attributable to a ligand-to-metal charge transfer (LMCT) transition and is the first example of a calcium-based MOF exhibiting blue-shifted fluorescence due to LMCT.

## Abstract 2

The solvothermal reaction of a mixture of calcium acetylacetonate and 1,4-naphthalenedicarboxylic acid (H_2_NDC) in a solution containing ethanol and distilled water gave rise to a metal–organic framework (MOF), {(H_3_O^+^)_2_[Ca(NDC)(C_2_H_5_O)(OH)]}_4_·1.1H_2_O.

## Graphical abstract

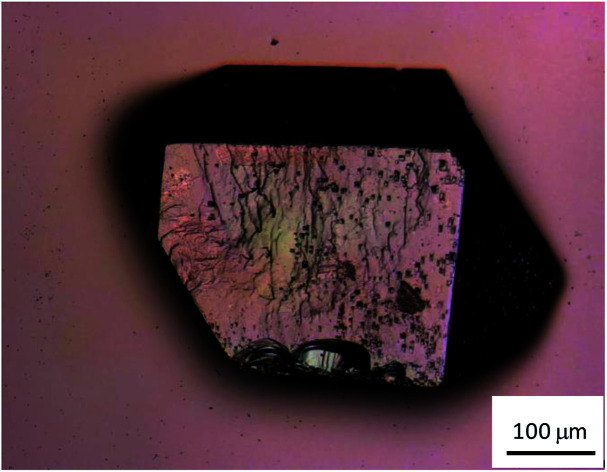

## 1. Introduction

Many conventional metal-complex porous materials collapse upon removal of the guest molecules.^1^ However, stable porous metal–organic frameworks (MOFs) composed of metal ions and organic linkers that do not collapse at room temperature were synthesized in the latter half of the 1990s,^2,3^ and subsequently there has been significant progress in the synthesis and functional analysis of porous materials. MOFs are used for several applications, such as the storage and separation of gas and for catalysis and magnetism.^4–13^ For example, MOFs with high adsorption capacity for hydrogen and methane have been reported.^14,15^

Fluorescent MOFs hold promise as sensors for detecting gases and toxic substances because they show strong, controllable fluorescence and have large surface areas with adjustable pore sizes.^16–25^ MOFs exhibit good fluorescence properties without requiring high temperature treatment because highly crystalline MOFs can be synthesized at relatively low temperatures.^26–28^ Among them, it is known that MOFs with Ca or Mg show various luminescence in the range from UV to Vis under UV/Vis excitation. In these MOFs, it is considered that they combined with organic ligands and exhibit new and interesting luminescence mechanism rather than luminescence due to the original d–d transition. However, researches or investigations for MOFs with Ca and Mg as luminescence materials are still few as compared with MOFs using Cu or Zn, and further development of these novel phosphors can be expected.

In this work, we prepared a CaNDC-MOF from calcium(ii) acetylacetonate and 1,4-naphthalenedicarboxylic acid (H_2_NDC) at low temperature. This MOF has a three-dimensional network structure with CaO_8_ coordination spheres composed of a calcium ion and carboxylates, and fluoresces due to a ligand-to-metal charge transfer (LMCT) transition.

## 2. Experimental

### Synthesis of the sample

We prepared CaNDC-MOF using a solvothermal synthesis method. Calcium(ii) acetylacetonate (Tokyo Chemical Industry Co., Ltd.) and H_2_NDC (Tokyo Chemical Industry Co., Ltd.) were mixed in an aqueous solution of ethanol (Wako Pure Chemical Industries, Ltd.) with stirring for 60 min in a polypropylene container. The molar ratio of the reagents was calcium(ii) acetylacetonate : H_2_NDC : distilled water : ethanol = 1 : 1 : 1000 : 1000. The mixed solution was placed in a vial and sealed, then kept in an oven at 100 °C for 7 days. After cooling to room temperature, the precipitate obtained was filtered and washed with ethanol, then dried in a vacuum for 2 days to obtain a white crystalline sample.

### Determination of the crystal structure

The crystal structure of CaNDC-MOF was determined by single-crystal X-ray diffraction (SXRD) measurement. The omega scanning technique was used to collect the reflection data using a Bruker D8 VENTURE goniometer with monochromatized MoK_α_ radiation operated at 40 kV and 40 mA. Data collection was carried out at low temperature (−183 °C) with flowing nitrogen gas. An initial structure of the unit cell was determined by a direct method using APEX2 software. The structural model was refined by a full-matrix least-squares method using SHELXL-2014/6 (Sheldrick, 2014). All calculations were performed using SHELXL programs.^29^ Visualization of the CaNDC-MOF was carried out using the Mercury and VESTA programs.^30^ The effective coordination numbers were calculated using the VESTA program. The solvent accessible voids were calculated using the Olex2 program^31^ with a 1.2 Å probe and a 0.2 Å grid size.

### Other analyses and instruments

Powder X-ray diffraction (PXRD) patterns were recorded on a Rigaku Ultima-IV X-ray diffractometer using CuK_α_ radiation at room temperature under ambient atmosphere. The data were collected angularly with 2*θ* of 5.0–55.0°, a step interval of 0.01°, and a scan speed of 2.00° min^−1^. The simulated PXRD patterns were obtained by using the Mercury program based on the single crystal data. Photographs of the sample were taken using a Keyence VK-9700 color 3D microscope and a 408 nm wavelength violet laser. Thermogravimetry-differential thermal analysis (TG-DTA) was carried out using a Shimadzu DTG-60AH instrument at a heating rate of 5 °C min^−1^ from room temperature to 600 °C with a nitrogen gas flow of 100 mL min^−1^. The samples were dried at 120 °C for 2 h and an empty test-tube was dried at 100 °C for 10 min under vacuum, then the nitrogen adsorption–desorption isotherm was measured using a BELSORP mini apparatus (microtrac BEL) operated at liquid nitrogen temperature, to obtain the BET specific surface area and nitrogen gas absorption–desorption characteristics. Fourier transform infrared (FT-IR) spectra were measured using a Horiba FT-720 instrument with a diamond attenuated total reflectance attachment at room temperature in the wavenumber range 4400 to 400 cm^−1^. Fluorescence and excitation spectra were recorded on a Jasco FP-6500 spectrophotometer with a xenon lamp in the wavelength range 200 to 800 nm at room temperature.

## 3. Results and discussion

**Fig. 1:**
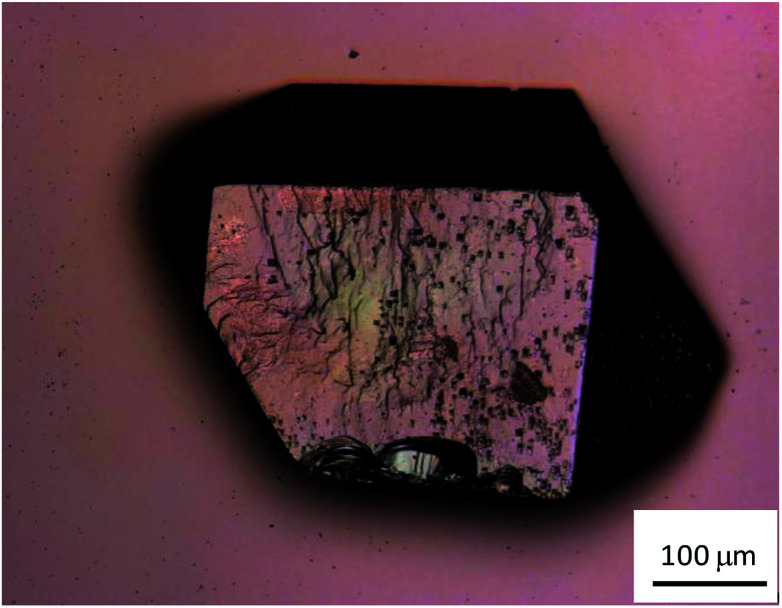
Laser scanning microscope image of CaNDC-MOF.

Fig. 1 shows a photograph of a CaNDC-MOF crystal grain taken using the laser scanning microscope. The crystal was colorless and fairly hard, with a diameter of about 150–200 μm.

The crystal surface was relatively smooth, but some asperities were found. The yield of CaNDC-MOF was about 74%.

**Fig. 2:**
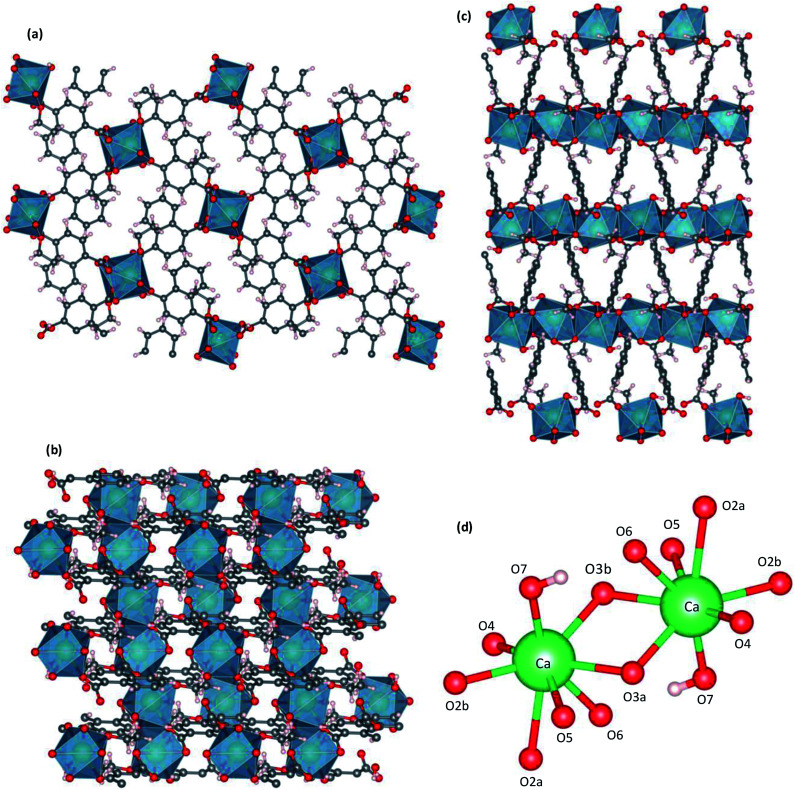
Crystal structure of CaNDC-MOF, viewed along *a* (a), *b* (b) or (c) *c* axis, and CaO_8_ structure (d).

Single-crystal X-ray structures of CaNDC-MOF are shown in Fig. 2 and detailed data are summarized in Table 1. CaNDC-MOF is monoclinic with the *P*2_1_/*n* space group, and the formula of CaNDC-MOF was obtained as C_56_H_48_Ca_4_O_24_. Since the formula weight (1265.28) of CaNDC-MOF is determined by SXRD after this weight loss, the 100 wt% formula weight is calculated to be 1581.25. The difference of about 164 corresponds to 9.1H_2_O. Finally, we decided the composition of CaNDC-MOF as {(H_3_O^+^)_2_[Ca(NDC)(C_2_H_5_O)(OH)]}_4_·1.1H_2_O by considering charge balance. Elemental analysis for hydrogen and carbon atoms was nearly satisfied (for CaNDC-MOF, {(H_3_O^+^)_2_[Ca(NDC)(C_2_H_5_O)(OH)]}_4_·1.1H_2_O, calculated [%]: C, 47.1; H, 4.6. Found [%]: C, 52.3; H, 4.5).

**Table 1:** Detailed data of CaNDC-MOF from SXRD measurement

Compound	CaNDC-MOF
Formula	C_56_H_48_Ca_4_O_24_
Formula weight	1265.28
Crystal system	Monoclinic
Space group	*P*2_1_/*n*
*a* (Å)	7.5660 (9)
*b* (Å)	17.062 (2)
*c* (Å)	11.3231 (15)
*α* (degree)	90
*β* (degree)	99.531 (4)
*γ* (degree)	90
*V* (Å^3^)	1441.5 (3)
*Z*	1
Temperature (K)	90.0
Effective coordination number	7.6205
Distance of Ca–O3b (Å)	2.3421
Ca–O2a (Å)	2.5612

**Fig. 3:**
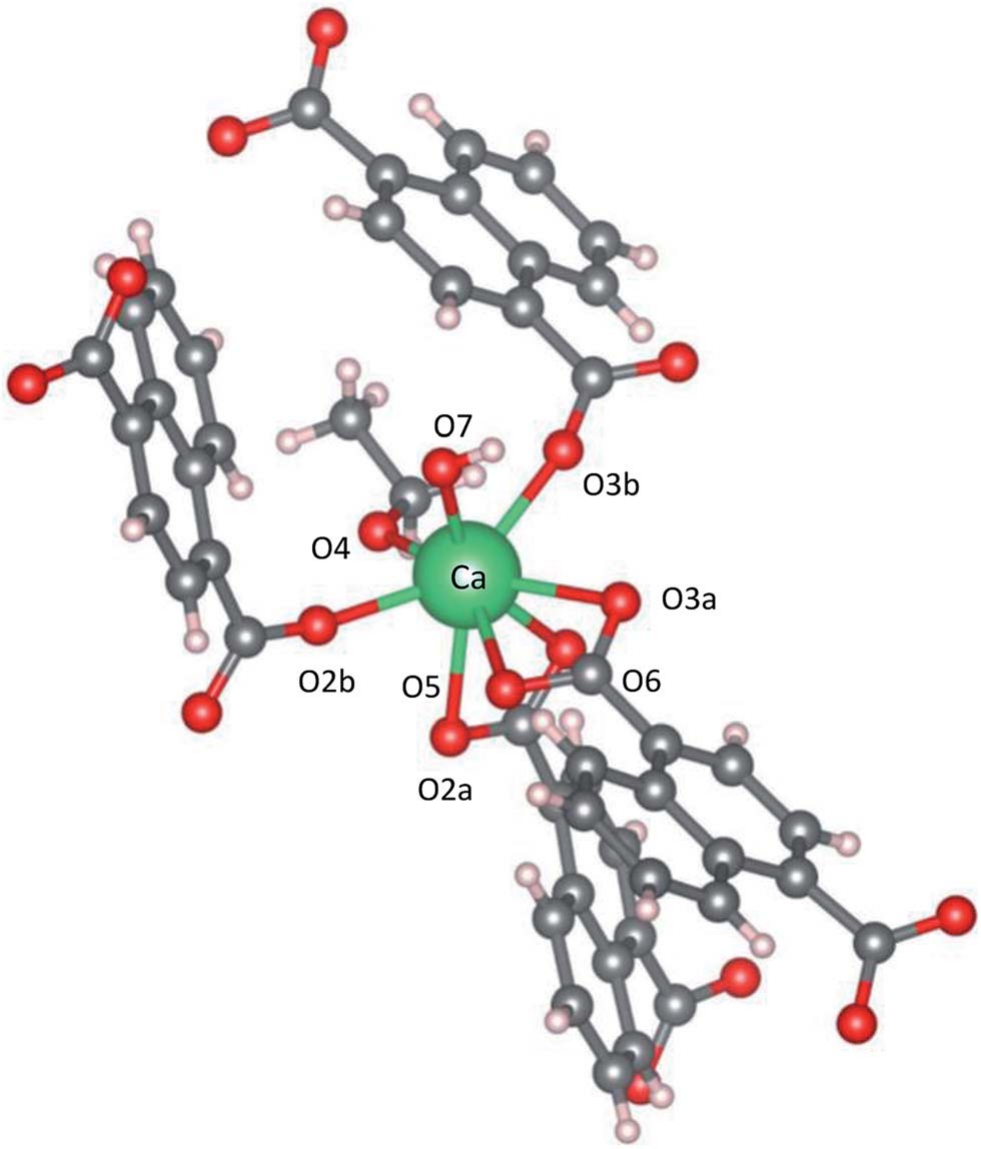
Coordination structure for a calcium atom.

Fig. 2 shows the three-dimensional network framework of CaNDC-MOF, comprising naphthalene rings and CaO_8_ coordination spheres. A pair of calcium atoms (green) are located along the *a*-axis direction at the center of a hexagon, six pairs of calcium atoms are at the vertices of the hexagon, and NDC ligands link the central pair with the vertex pairs (Fig. 2a). There are four void-linkage lines within the hexagon. However, the structure may have higher density along the *b*-axis direction (Fig. 2b). The CaO_8_ coordination spheres form two-dimensional layer structures that are connected by NDC ligands to create trapezoid voids along the *c*-axis direction (Fig. 2c). A calcium atom in CaNDC-MOF has an eight-coordinated structure with four NDC ligands (Fig. 3). Six oxygen atoms (O2a, O2b, O3a, O3b, O5 and O6) coordinated to a calcium atom are part of the carboxyl groups of the NDC ligands. However, one oxygen atom (O4) is found to be in an ethoxy group and another (O7) is supplied from the solvent. The presence of this ethoxy group was verified by FT-IR and is likely derived from ethanol in the solvent. The CaO_8_ coordination spheres with the nominal coordination number for a calcium ion of 8 are connected with each other through two oxygen atoms of the carboxyl groups. In order to investigate the coordination environment further, the effective coordination number (ECoN) of CaNDC-MOF was calculated to be 7.6205 for calcium.^32,33^ The calculated ECoN is slightly lower than the nominal ECoN value of 8 because the longest bond (for Ca–O2a) is about 10% longer than the shortest bond (for Ca–O3b). We also calculated the bond valence values using the VESTA program to be −0.2194 for Ca–O2a and −0.3967 for Ca–O3b, which indicate probable weak interactions between these calcium and oxygen atoms.

**Fig. 4:**
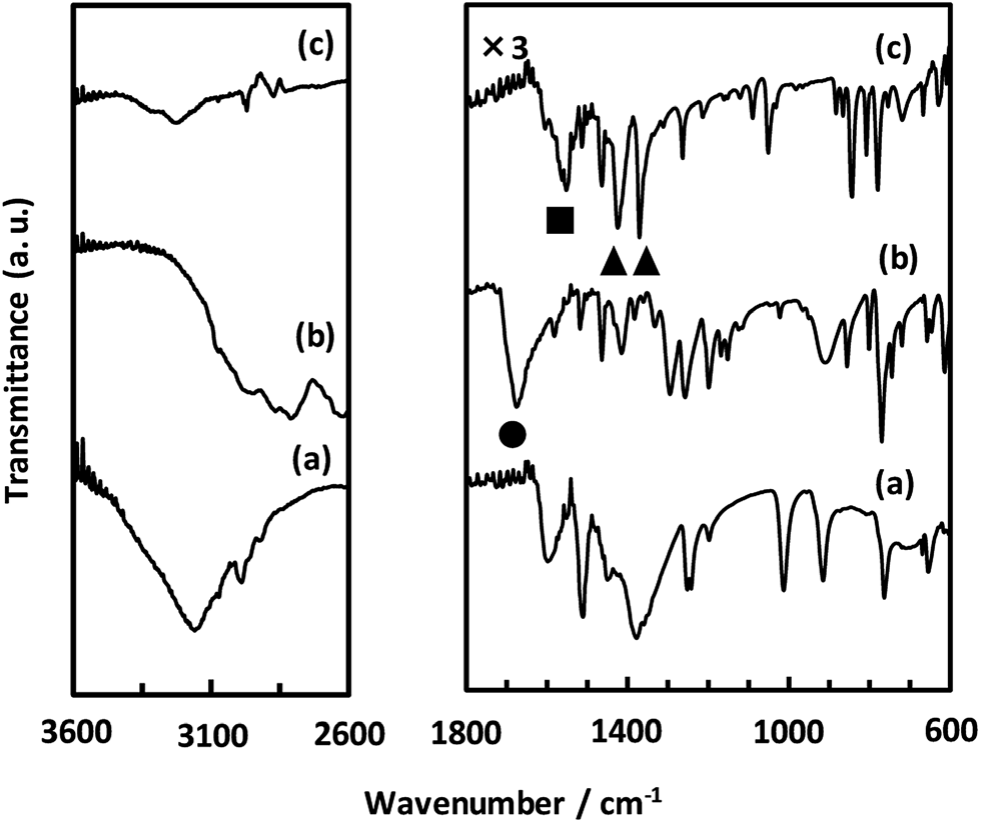
FT-IR spectra of calcium(ii) acetylacetonate (a), H_2_NDC (b) and CaNDC-MOF (c).

As described above, we examined the structure of CaNDC-MOF and identified the ethoxy group by FT-IR spectroscopy. Fig. 4 shows the FT-IR spectra of calcium(ii) acetylacetonate, H_2_NDC and CaNDC-MOF. In the absence of an ethoxy group but in the presence of ethanol, an alcohol peak should be seen in the FT-IR spectra. However, no broad peak around 3600 cm^−1^ assignable to the *ν*(O–H) of alcohol was observed in CaNDC-MOF. Instead, two peaks, one each at 1470 and 1380 cm^−1^ (▲), are observed and attributed to the *δ*(–CH_2_) and *δ*(–CH_3_), respectively, of an ethoxy group in CaNDC-MOF,^34^ thereby confirming the presence of an ethoxy group in CaNDC-MOF. Next, we examined the arrangement of the protons. No broad peak at 2600–3400 cm^−1^ attributed to the *ν*(O–H) of carboxylic acid was observed in CaNDC-MOF, and a peak due to *ν*(C=O) vibration at 1670 cm^−1^ in H_2_NDC (●) was shifted to 1500–1600 cm^−1^ in CaNDC-MOF (■), confirming deprotonation of the NDC molecules.

Broad peaks at 2800–3200 cm^−1^ are attributed to hydrogen bonding networks and are thought to be affected by the distance between the O–O atoms. This hydrogen bond is due to water molecules present irregularly in the voids of the crystal.^35^ It is difficult to determine the positions of these water molecules by crystal structure analysis, but this result is consistent with the TG-DTA results described below.

**Fig. 5:**
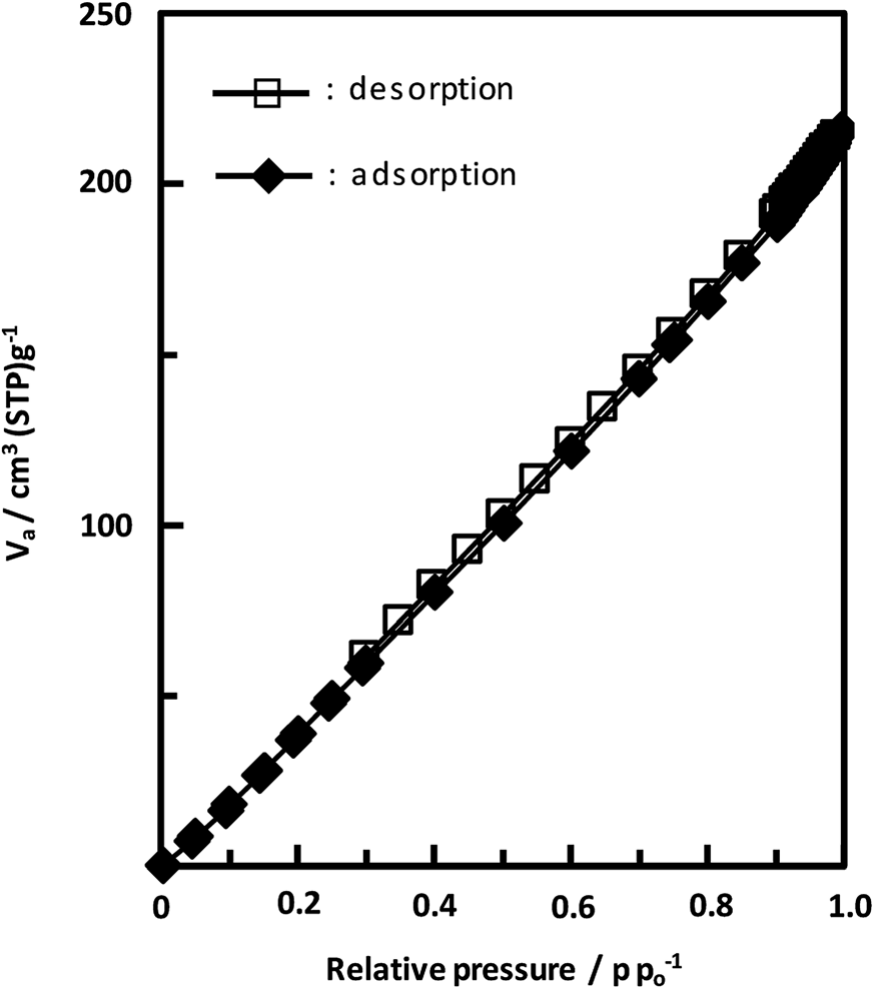
Adsorption and desorption isotherms of CaNDC-MOF for using nitrogen gas.

**Fig. 6:**
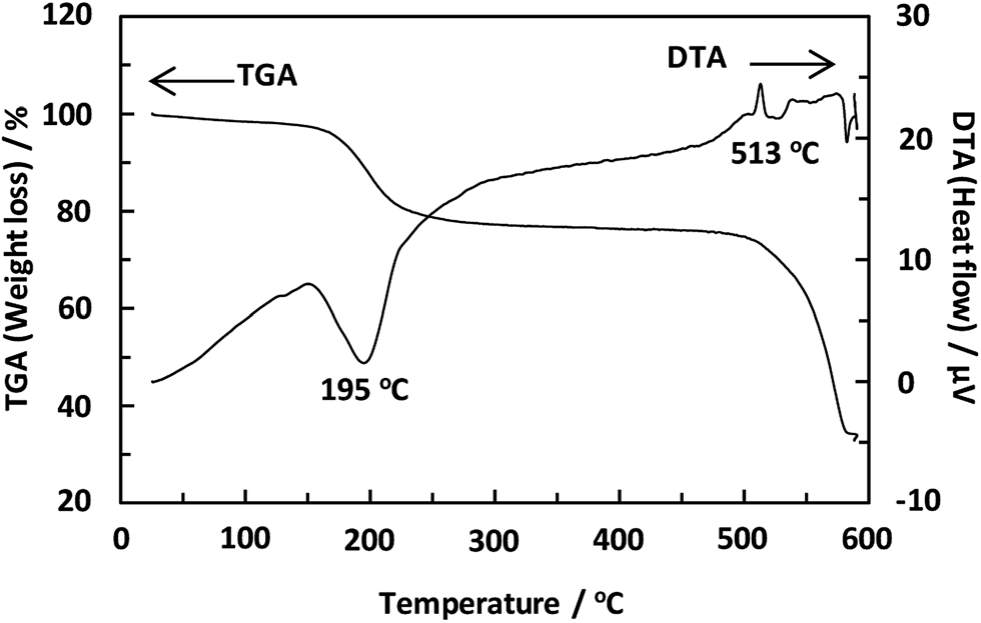
TGA and DTA curves of CaNDC-MOF.

**Fig. 7:**
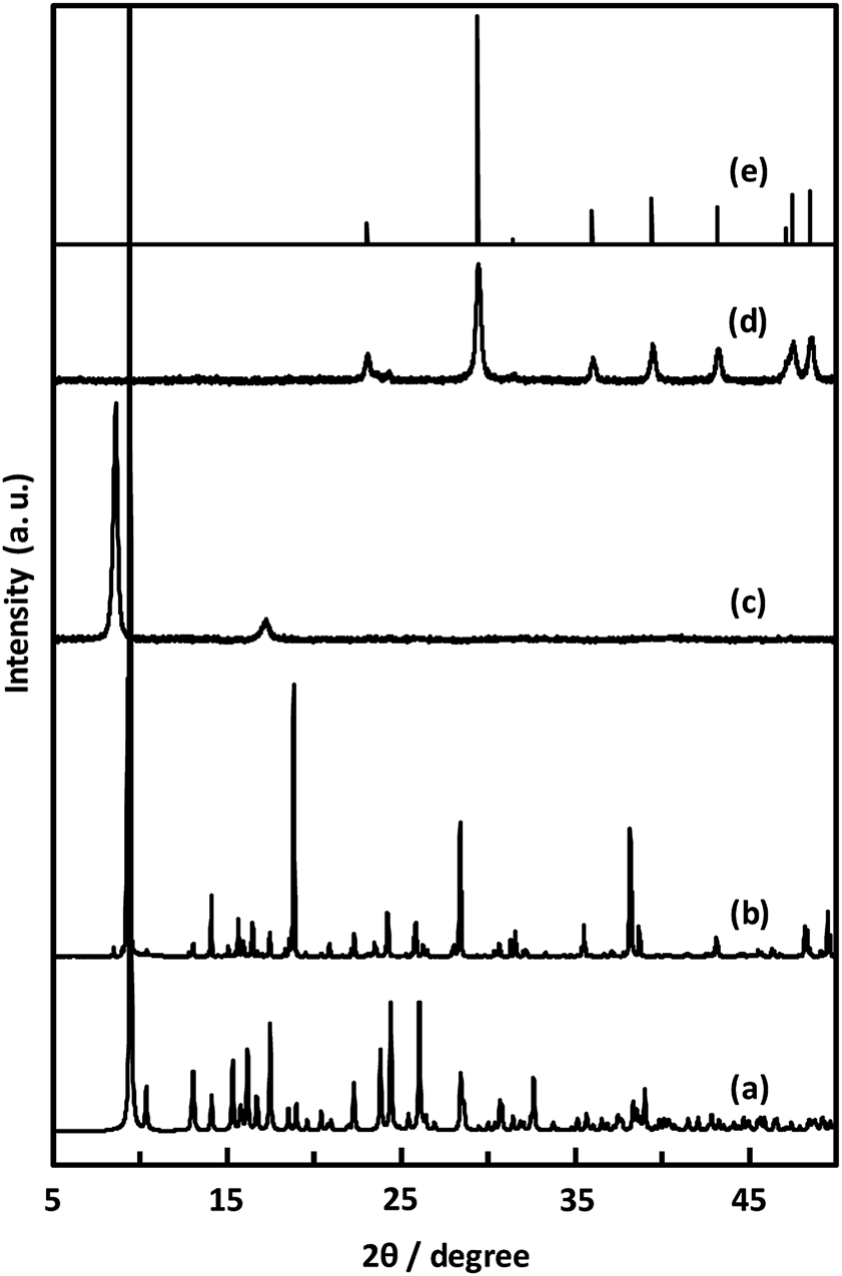
PXRD patterns of simulated (a), CaNDC-MOF (b), CaNDC-MOF after being heated at 250 (c) and 600 °C (d), and CaCO_3_ standard pattern (e).

Unexpectedly, we observed a Henry-type adsorption isotherm (Fig. 5), indicating that CaNDC-MOF has small voids with ultramicropore volumes. We calculated a pore volume of 0.322 cm^3^ g^−1^ using the Dubinin–Astakhov method. We also calculated a solvent accessible volume of 12.9 Å^3^ from the structural data obtained without drying the sample. CaNDC-MOF has slightly accessible channels (BET surface area: 353 m^2^ g^−1^), most probably caused by small cracks in the crystal structure formed during the drying procedure required for degassing. The TGA and DTA curves for CaNDC-MOF in Fig. 6 show an endothermal peak at 195 °C and a weight loss of about 20 wt% at this temperature. In addition, an exothermic peak was observed at 513 °C with a large weight loss. We investigated these peaks by measuring the PXRD patterns of CaNDC-MOF heated at 250 and 600 °C, and the results are shown in Fig. 7. The structure of CaNDC-MOF heated at 250 °C collapsed, and thus the endothermal peak at 195 °C (Fig. 6) is attributed to destruction of the structure due to reaction of the organic components of the complex or to the evaporation of crystal water. The sample heated at 600 °C produced calcium carbonate (PDF no. 00-47-1743), and thus the exothermic peak at 513 °C (Fig. 6) arised from combustion of the organic components and the formation of calcium carbonate.

**Fig. 8:**
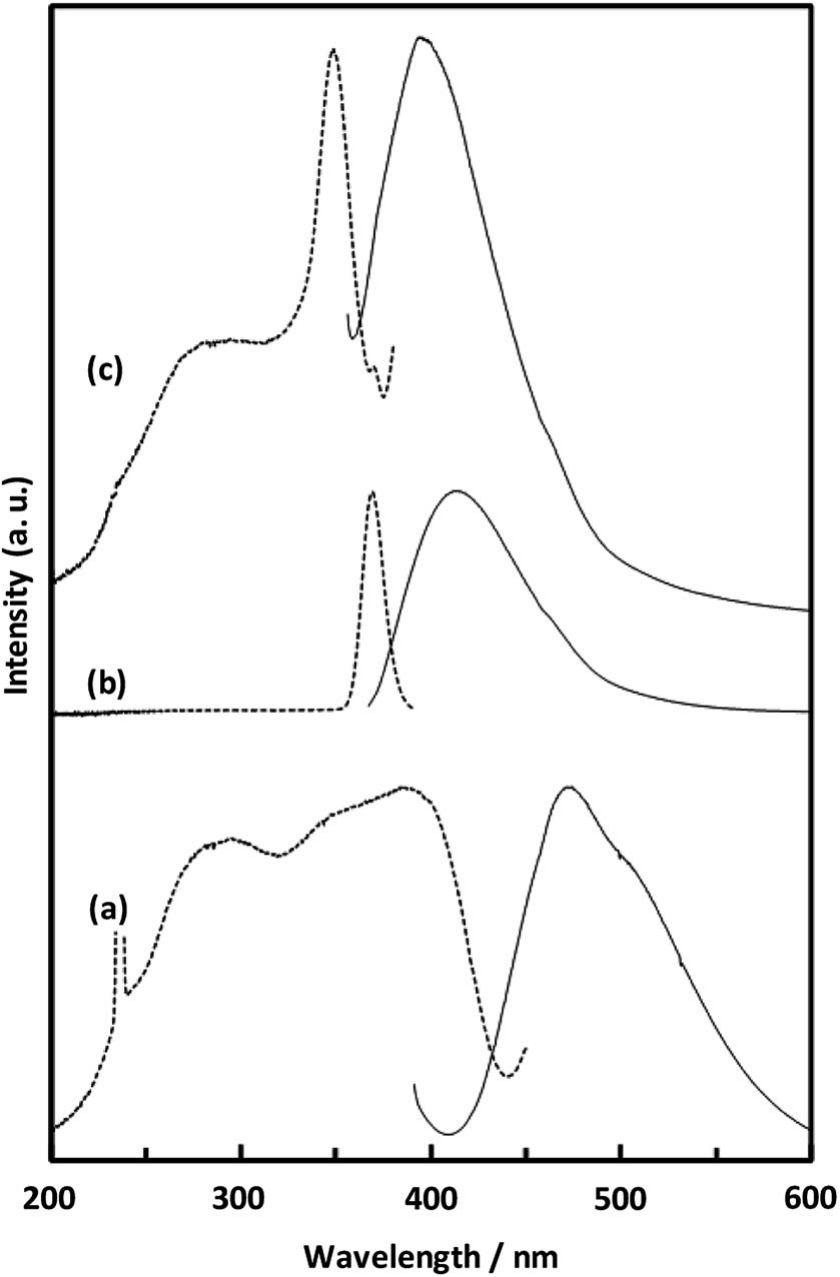
Solid-state fluorescence (solid lines) and excitation (broken lines) spectra of H_2_NDC powder (a), H_2_NDC dissolved in water/ethanol (b), and CaNDC-MOF (c).

Fig. 8 shows the fluorescence and excitation spectra of CaNDC-MOF and of H_2_NDC. The NDC ligand gives a broad fluorescence peak at 470 nm when excited at 385 nm, attributable to the π* → n transition.^36,37^ CaNDC-MOF showed violet fluorescence with a broad peak at 395 nm when excited at 350 nm. Comparing these results, the position of the maximum fluorescence peak of CaNDC-MOF is 75 nm shorter than that of the NDC ligand, and the difference in the maximum excitation wavelength is 35 nm.

There are two suggestions for the origin of fluorescence in CaNDC-MOF. Firstly, this blue-shifted fluorescence can be assigned to the LMCT transition,^38,39^ where calcium and the NDC ligand bond to each other to widen the energy gap. This blue-shift shows that H_2_NDCs are not clumped but rather integrated into the structure of CaNDC-MOF, and the distance between the molecules lengthens. LMCT-based fluorescence has been reported for Zn and Cd cation MOFs, which shows significant red-shifts compared with the fluorescence of the ligands.^40–42^ To our knowledge, this is the first research of a calcium MOF showing a blue-shifted LMCT, although there have been several reports of red-shifted fluorescence.^43^

Secondly, it is considered that the origin of fluorescence of Ca-MOF is just only from the organic ligands, and Ca has no relationship. H_2_NDC in a water/ethanol mixed solution (H_2_NDC : water : ethanol = 1 : 1000 : 1000) showed its maximum fluorescence peak at 412 nm under excitation at 369 nm. These excitation and fluorescence spectra are similar to those of Ca-MOF. A conceivable reason for this similarity is described as follows: in the solid crystalline state Ca-MOF, the ligands are dispersed to the same extent as in the solution, and they differ from the fluorescence of the aggregated state like solid ligands. Further study is needed to clarify the fluorescence mechanism.

Anyway, our finding suggests that the calcium atom is incorporated into the structure of the MOF, leading to various fluorescence applications.

## 4. Conclusions

We succeeded in the synthesis of a calcium-based metal–organic framework (CaNDC-MOF) using a solvothermal method at 100 °C. Moreover, CaNDC-MOF possesses an ethoxy group obtained from the solvent and a unique 2D layer network structure with voids. CaNDC-MOF shows blue-purple fluorescence which is blue-shifted by 75 nm compared with the fluorescence of the free organic linker H_2_NDC. This blue-shift may appear to be due to resulting from the incorporation of calcium atoms and NDC ligands into the MOF structure.

## Conflicts of interest

There are no conflicts to declare.

## Supplementary Material

RA-008-C8RA06043F-s001
